# Supplementary material for: Characterization of Acid-Aged Biochar and Its Ammonium Adsorption in an Aqueous Solution
Source: Materials (Basel). 2020 May 14;13(10):2270. doi: 10.3390/ma13102270 (PMC7287775; doi:10.3390/ma13102270)
Supplement: Supplementary file 1 [file materials-13-02270-s001.pdf]

materials

Supplementary Materials

Characterization of Acid-Aged Biochar and its Ammonium Adsorption in an Aqueous Solution

Zhiwen Wang [1,2], Jie Li [2,*], Guilong Zhang [2], Yancai Zhi [2], Dianlin Yang [2], Xin Lai [2] and Tianzhi Ren [1,2,*]

[1] College of Resources and Environment, Northeast Agricultural University, Harbin 150030, China; wangzhiwen612@163.com

[2] Agro-Environmental Protection Institute, Ministry of Agriculture and Rural Affairs, Tianjin 300191, China; zhiyancai@126.com (Y.Z.); zhangguilong@caas.cn (G.Z.); yangdianlin@caas.cn (D.Y.); laixin@caas.cn (X.L.)

* Correspondence: lijie@caas.cn (J.L.); rentianzhi@caas.cn (T.R.); Tel: +86-22-23611819 (J.L.); Fax: +86-22-23611802 (J.L.)

Received: 12 March 2020; Accepted: 9 May 2020; Published: 14 May 2020

Table S1. Pore structure of the original and acid modified biochar.

Samples	S_{DFT} ($m^2 \cdot g^{-1}$)	V_{DFT} ($cc \cdot g^{-1}$)	S_{BJH} ($m^2 \cdot g^{-1}$)	V_{BJH} ($cc \cdot g^{-1}$)	D_P (nm)
PBC	322.703	0.088	0.365	0.006	14.882
H_2O-PBC	358.505	0.097	0.607	0.006	9.837
H_2SO_4-PBC	382.557	0.105	0.077	0.001	10.234
HCl-PBC	357.567	0.098	0.111	0.001	10.454
H_2O_2-PBC	378.470	0.103	0.741	0.010	14.968

Table S2. Surface elemental composition of the original and acid modified biochar.

Sample	Surface elemental composition[a] (%)								
	C	O	N	Si	Ca	Mg	S	O/C	(O+N)/C
PBC	84.09	13.57	0.85	0.35	0.53	0.17	0.19	16.14	17.15
H_2O-PBC	83.08	13.64	1.02	0.87	0.59	0.57	0.15	16.42	17.65
HCl-PBC	83.99	13.46	1.30	0.88	ND	ND	0.18	16.03	17.57
H_2SO_4-PBC	84.24	13.28	1.32	0.88	ND	ND	0.18	15.77	17.33
H_2O_2-PBC	81.21	14.36	1.48	1.40	0.83	0.57	ND	17.68	19.50

[a] Estimated on an ash free basis

Table S3. Elemental composition of the original and acid modified biochar.

Sample	Bulk elemental composition[a] (%)						
	C	H	O	N	S	H/C[b]	O/C[b]
PBC	71.86	2.84	14.54	2.32	N.D. (<0.3)	3.95×10^{-2}	2.02×10^{-1}
H_2O-PBC	75.35	2.83	14.60	2.10	N.D. (<0.3)	3.76×10^{-2}	1.94×10^{-1}

HCl-PBC	74.76	2.75	13.64	2.13	0.40	3.68×10^{-2}	1.82×10^{-1}
H_2SO_4-PBC	73.28	2.80	15.01	2.38	N.D. (<0.3)	3.87×10^{-2}	2.21×10^{-1}
H_2O_2-PBC	73.50	2.74	13.71	2.54	0.38	3.73×10^{-2}	1.87×10^{-1}

[a] Estimated on an ash free basis; [b] Estimated on a mass/mass basis.

Table S4. (a) The Chemical bond composition of biochar (C1s).

Sample	XPS C1s chemical bond composition (%)			
	C-H,C-C,C=C	C-O	C=O	O-C=O
PBC	56.3	25.6	11.8	6.2
H_2O-PBC	55.3	15.9	16.6	12.3
HCl-PBC	54.3	18.1	15.2	12.4
H_2SO_4-PBC	53.6	19.0	16.5	10.8
H_2O_2-PBC	50.5	22.6	15.8	11.1

Table S4. (b) The Chemical bond composition of biochar (O1s, N1s).

Sample	O1s Chemical bond composition (%)			N1s Chemical bond composition (%)	
	C=O	O-C=O	O-H/-COOR	Pyrrolic-N	Pyridinic-N
PBC	42.0	37.5	20.5	77.7	22.3
H_2O-PBC	45.2	38.4	16.4	82.7	17.3
HCl-PBC	45.2	28.0	26.7	74.1	25.9
H_2SO_4-PBC	41.1	33.9	25.0	71.5	28.5
H_2O_2-PBC	42.1	32.0	25.9	73.7	26.3

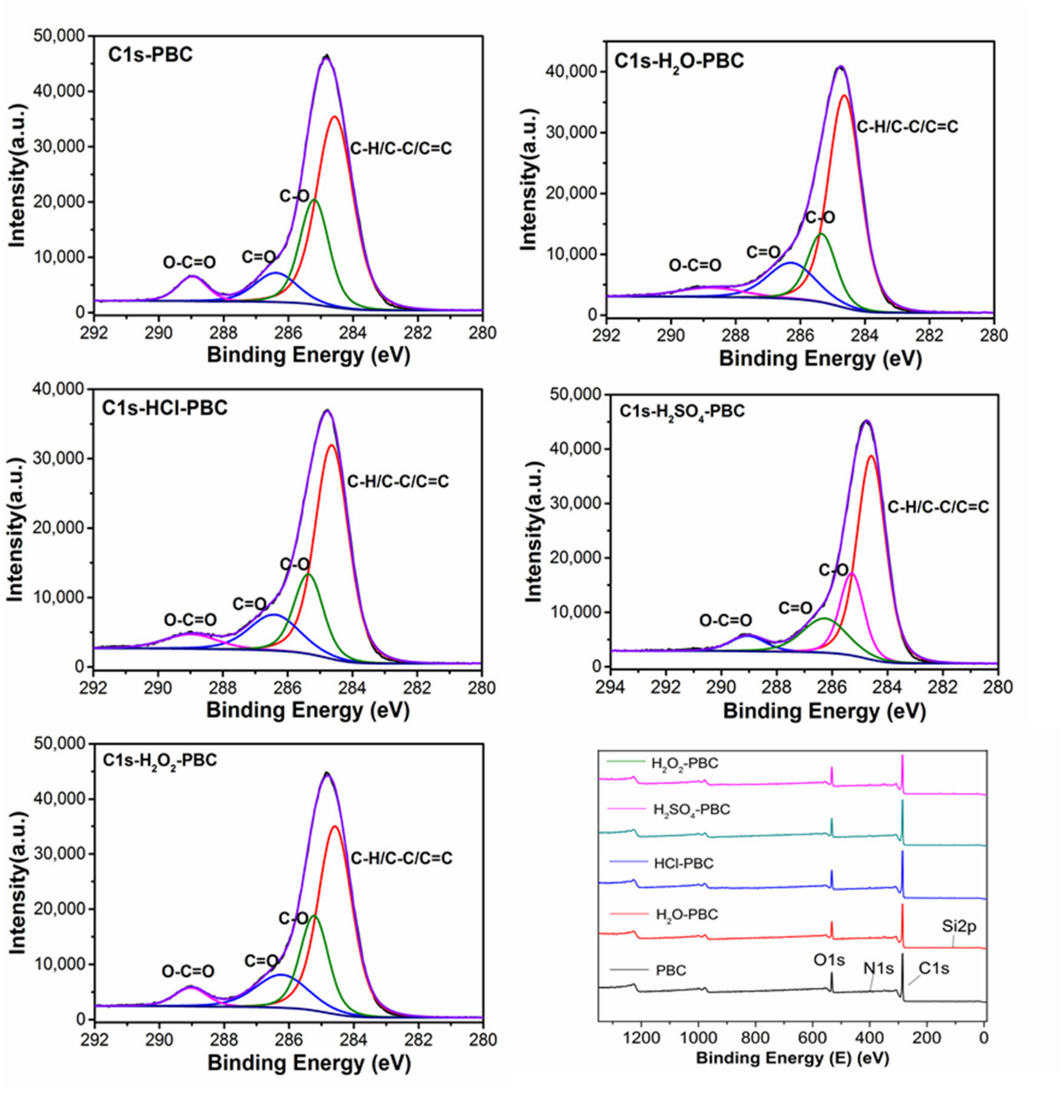

Figure S1. The XPS spectra of biochar and their C1s XPS-peak-differentation-imitating.

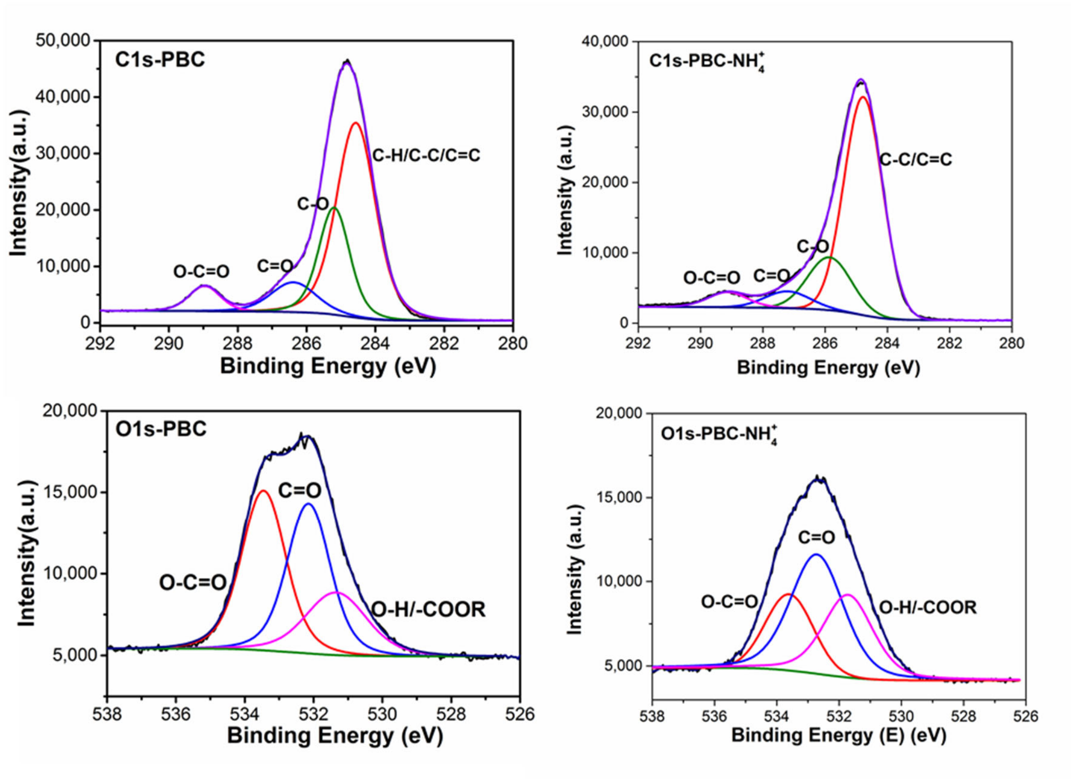

Figure S2. XPS spectra of C1s and O1s of PBC before and after NH_4^+-N adsorption.

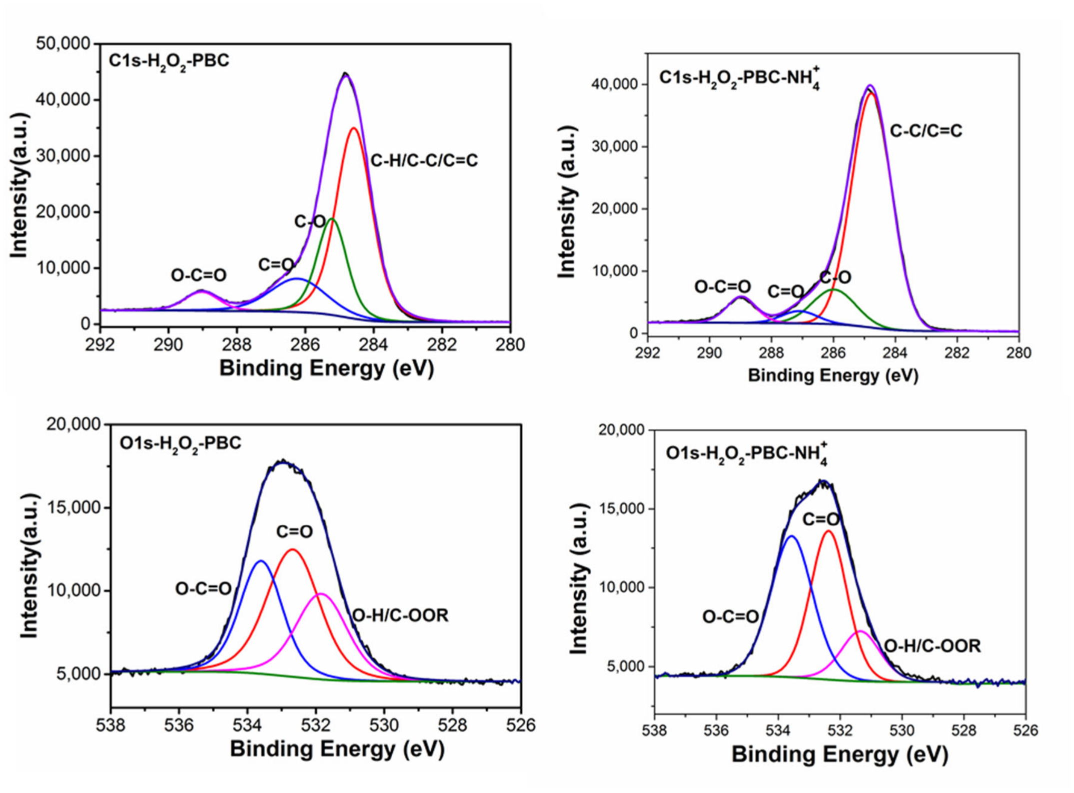

Figure S3. XPS spectra of C1s and O1s of H_2O_2-PBC before and after NH_4^+-N adsorption.

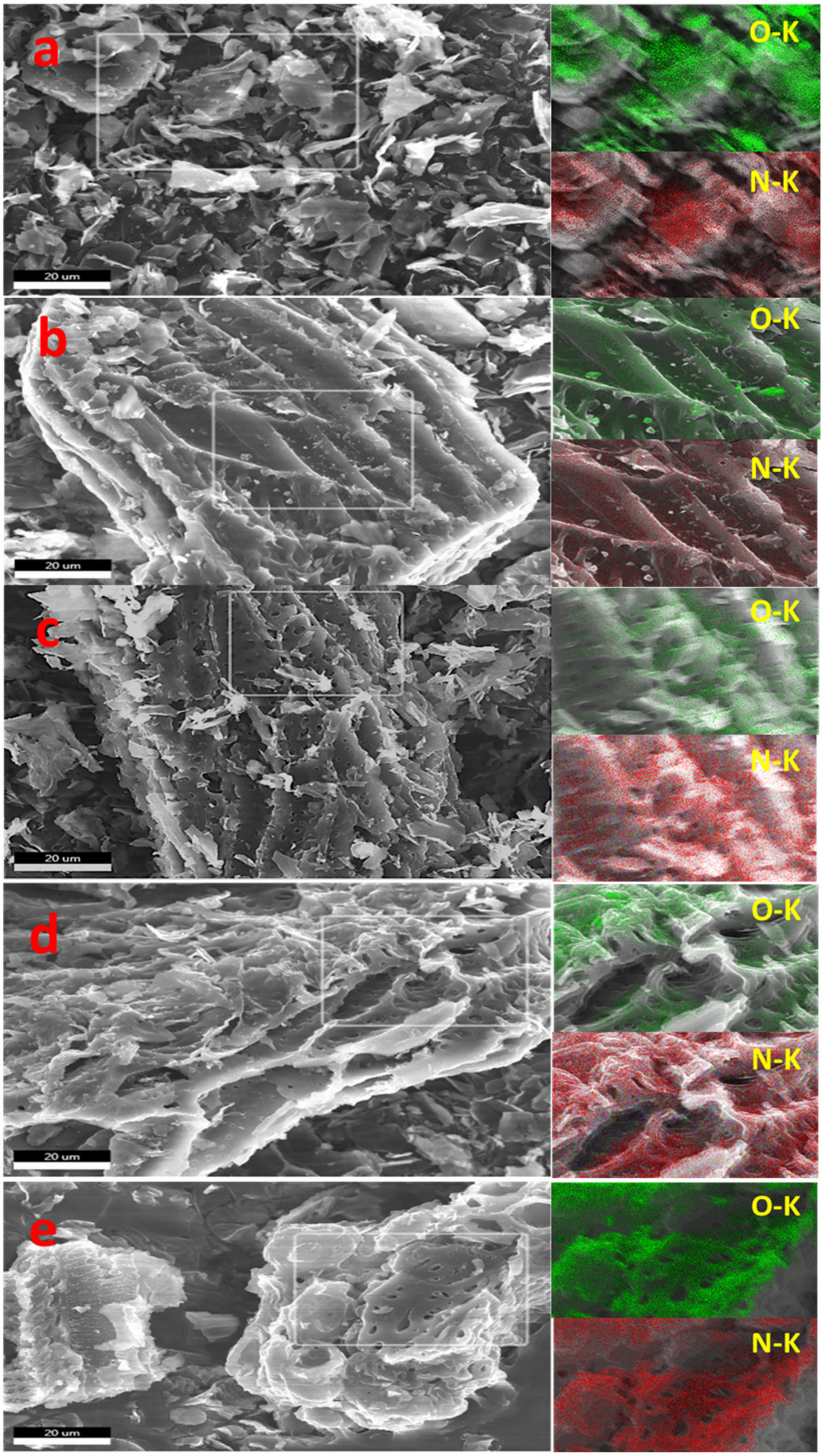

Figure S4. SEM and EDS images of the five biochars. (**a,b,c,d,e** represent the adsorption of NH_4^+ before and after PBC, H_2O-PBC, HCl-PBC, H_2SO_4-PBC, and H_2O_2-PBC, respectively.

© 2020 by the authors. Submitted for possible open access publication under the terms and conditions of the Creative Commons Attribution (CC BY) license (http://creativecommons.org/licenses/by/4.0/).
